# An analysis of respiratory induced kidney motion on four-dimensional computed tomography and its implications for stereotactic kidney radiotherapy

**DOI:** 10.1186/1748-717X-8-248

**Published:** 2013-10-26

**Authors:** Shankar Siva, Daniel Pham, Suki Gill, Mathias Bressel, Kim Dang, Thomas Devereux, Tomas Kron, Farshad Foroudi

**Affiliations:** 1 Department of Radiation Oncology, Peter MacCallum Cancer Centre, Melbourne, Australia; 2 Sir Peter MacCallum Department of Oncology, University of Melbourne, Melbourne, Australia; 3 Radiation Therapy Services, Peter MacCallum Cancer Centre, Melbourne, Australia; 4 Department of Biostatistics and Clinical Trials, Peter MacCallum Cancer Centre, Melbourne, Australia; 5 Department of Physical Sciences, Peter MacCallum Cancer Centre, Melbourne, Australia; 6 Peter MacCallum Cancer Centre, Locked Bag 1, A’Beckett Street, East Melbourne, Victoria 8006, Australia

**Keywords:** Kidney, Stereotactic, 4DCT, Respiratory motion, Radiation, ITV margin

## Abstract

**Background and purpose:**

Stereotactic ablative body radiotherapy (SABR) is an emerging treatment modality for primary renal cell carcinoma. To account for respiratory-induced target motion, an internal target volume (ITV) concept is often used in treatment planning of SABR. The purpose of this study is to assess patterns of kidney motion and investigate potential surrogates of kidney displacement with the view of ITV verification during treatment.

**Material and methods:**

Datasets from 71 consecutive patients with free breathing four-dimensional computed tomography (4DCT) planning scans were included in this study. The displacement of the left and right hemi-diaphragm, liver dome and abdominal wall were measured and tested for correlation with the displacement of the both kidneys and patient breathing frequency.

**Results:**

Nine patients were excluded due to severe banding artifact. Of 62 evaluable patients, the median age was 68 years, with 41 male patients and 21 female patients. The mean (range) of the maximum, minimum and average breathing frequency throughout the 4DCTs were 20.1 (11–38), 15.1 (9–24) and 17.3 (9–27.5) breaths per minute, respectively. The mean (interquartile range) displacement of the left and right kidneys was 0.74 cm (0.45-0.98 cm) and 0.75 cm (0.49-0.97) respectively. The amplitude of liver-dome motion was correlated with right kidney displacement (r=0.52, p<0.001), but not with left kidney displacement (p=0.796). There was a statistically significant correlation between the magnitude of right kidney displacement and that of abdominal displacement (r=0.36, p=0.004), but not the left kidney (r=0.24, p=0.056). Hemi-diaphragm displacements were correlated with kidney displacements respectively, with a weaker correlation for the left kidney/left diaphragm (*r=*0.45, [95% CI 0.22 to 0.63], *p*=<0.001) than for the right kidney/right diaphragm (*r*=0.57, [95% CI 0.37 to 0.72], *p*=<0.001).

**Conclusions:**

For the majority of patients, maximal left and right kidney displacement is subcentimeter in magnitude. The magnitude of kidney motion cannot be reliably estimated from the diaphragmatic, liver dome or abdominal wall surrogates. One explanation may be that the kidneys are not uniformly in contact with the surrogates investigated in this study. Further investigation is required before surrogates of kidney displacement are used for clinical SABR delivery.

## Introduction

Renal cell carcinoma (RCC) is one of the 10 most common cancers in men and women, with incidence rates increasing by 4.1% per year in men and 3.3% per year in women between 2004 and 2008 [1]. Whilst surgery is the standard of care for primary disease [2], stereotactic ablative body radiotherapy (SABR) has emerged as potentially curative treatment approaches for patients who refuse or are unsuitable for surgery. A recent systematic review reports that local control rates from SABR in primary RCC are excellent, ranging from 84%-100% [3]. However, given the potential risk of severe toxicity to surrounding normal organs susceptible to hypofractionated radiotherapy (such as small bowel), significant consideration must be given to limit dose to normal tissue. One strategy to limit risk is to utilize rigorous image guidance methods for SABR delivery. Integral to this aim is the ability to mitigate geometric uncertainty arising from the target kidney motion.

A major challenge in the image guidance process is intrafractional kidney displacement due to respiration. Renal tumours are often of similar density to the surrounding normal kidney and can be difficult to visualize using cone beam CT (CBCT). The use of contrast enhancing agents is often contraindicated by the pre-existing renal dysfunction that is prevalent in this patient population. In light of challenging imaging conditions, one potential strategy to account for kidney motion is treatment planning using the internal target volume (ITV) concept [4]. In order to validate the appropriateness of ITV margins constructed at planning, surrogates of tumor displacement can be matched at the time of treatment delivery. These surrogates include the diaphragm as visualized using fluoroscopic kilovoltage (kV) imaging, or external markers placed on the abdominal wall. The use of external markers is often favored due to the advantages of real-time tracking and a reduction in undesirable excess ionizing radiation to the patient [5]. When using 3D on board imaging with CBCT, a novel and as yet unexplored potential surrogate is the excursion of the liver dome. However, the validity of the use of any surrogate of kidney position is predicated on validating a robust relationship between the kidneys and surrounding organ motion.

The purpose of this study is to assess the relationship of kidney displacement with clinically relevant surrogates that can be easily measured at the time of the treatment planning CT. The surrogates investigated in this study include the anterior abdominal wall, the diaphragms, and the probability density function (PDF) of the liver dome (previously described by Guckenberger et al. [6]). These relationships have implications for radiotherapy planning and treatment delivery, as typical SABR plans using an ITV concept employ a very narrow margin to the final planning target volume (PTV). As such, these plans rely heavily on accurate quantification of both target position and displacement. We investigated if the displacement of these surrogates can be used to confirm the magnitude of displacement of the kidney and therefore serve to validate the appropriateness of the ITV margin used for kidney SABR. The central hypothesis of this study is that the magnitude of kidney displacement correlates to the PDF width.

## Materials and methods

This is a retrospective study of 71 consecutive patients who had 4DCT planning scans including partial or full view of the left and right kidneys at a single institution. Nine patient datasets were excluded due to excessive banding artifact. Of the remaining 62 patients, 53 had thoracic tumors and 9 patients had liver tumors. The median age was 68 years, with 41 male patients and 21 female patients. This study had independent review board (IRB) approval at the Peter MacCallum Cancer Centre.

Patients were scanned in the arms up position under relaxed free-breathing conditions without any compression devices. A respiratory sorted four-dimensional computed tomography (4DCT) dataset was generated using the Philips Brilliance® CT scanner coupled with a Philips Bellows system® as surrogate marker for breathing phase (Philips Medical Systems, Best, The Netherlands). The bellows system consists of an elasticised belt worn around the abdomen that expands and contracts with respiratory motion. A pressure transducer converts the variation of pressure in the bellows into a voltage signal, which is digitized and transmitted to the CT scanner. The resultant data is presented as a trace demonstrating respiratory motion and calculated number of breaths per minute. The calculated respiratory rate is used to select an appropriate pitch for couch motion during CT scanning. Typically, the respiratory trace was observed for a period of approximately one minute to ensure inter-cycle stability prior to CT acquisition. The CT scanner was commissioned to acquire 4DCT scans in helical mode, and to bin the CT slices into 10 phases for image reconstruction. The patients were imaged using 140 kVp, 3 mm slice thickness, 3 mm increment, and 0.44 s rotation time, and images were reconstructed with ~3.5 mm^3^ voxel resolution (3 mm slice thickness × 1.0742 mm pixel spacing). The 4DCT scan acquisition was approximately 90 seconds in duration, typically incorporating information from 18–27 patient breaths. From the respiratory-sorted imaging datasets, the maximum expiration and inspiration phases were identified and subsequently exported to the treatment planning system for analysis. This methodology cannot account for irregularity of the breathing cycle. However, in most motion management techniques, including the Internal Target Volume delineation used in our centre, the accurate identification of the extremes of breathing is of greater importance.

**Figure 1:**
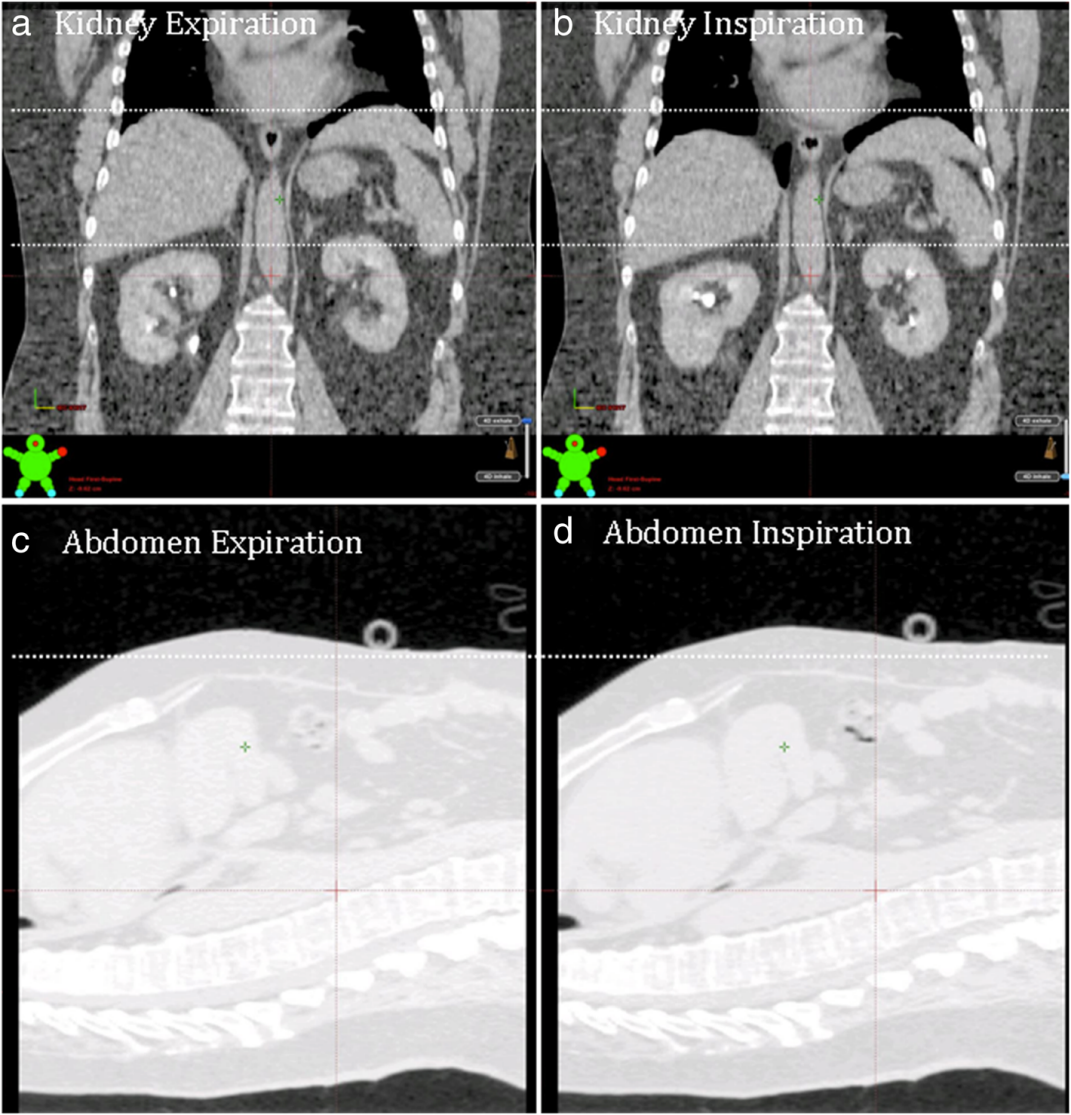
**Measurement of kidney and abdominal displacement.** Panels **a)** and **c)** show the patient dataset in maximum expiration, panels **b)** and **d)** show the patient dataset in maximum inspiration.

All measurements were performed on an Eclipse® workstation v 8.9 (Varian Medical Systems, Palo Alto, CA) using standardized abdominal window/level settings. Left and right diaphragm displacement was measured using a predefined coronal reference plane: The most anterior aspect of the T12 vertebral body, at midline and mid-vertebral height. Cranio-caudal kidney apex displacements were viewed across axial, sagittal and coronal CT for the maximum displacement (Figure 1). The magnitude of displacement of the abdominal wall was measured in the ventero-dorsal plane. The magnitude of displacement was defined as the difference between maximum and minimal organ position throughout all viewed datasets. To quantify liver dome displacement, a line profile tool was used to display and visualize the liver dome’s probability density function (PDF) at the coronal reference plane. This measurement was performed on the 4DCT reconstructed *averaged* CT dataset (which is a composite of all 10 respiratory phases, equivalent of a slow CT scan). A line profile tool was used to measure the PDF width displaying the Hounsfield Unit variation due to liver dome motion. This technique was adapted from that previously described [6] to measure liver excursion in CBCT datasets during online image guidance of SABR.

**Figure 2:**
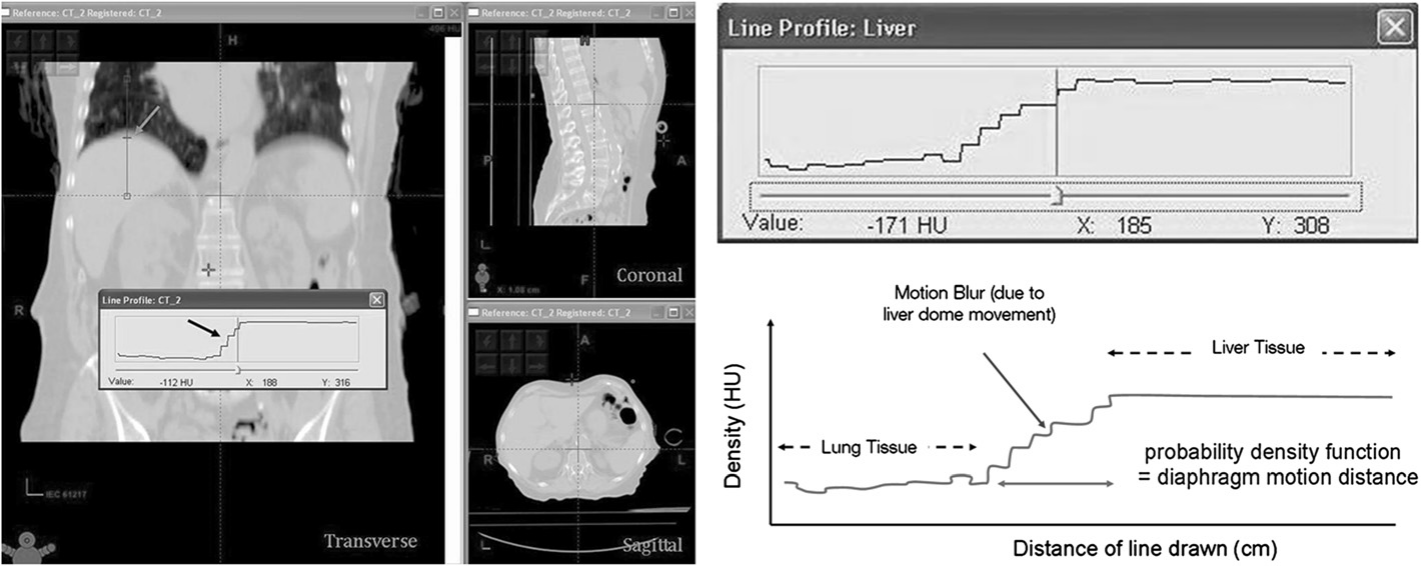
Measurement of probability density function.

A Pearson correlation coefficient was subsequently used to test the liver dome PDF width for correlation with the displacement of kidneys and abdominal wall (Figure 2). The width of the PDF, and right and left kidneys were also correlated to the breathing frequency from each recorded breathing trace recorded from the 4DCT scans using a Pearson statistic.

## Results

Under free breathing conditions the mean displacement as identified on 4DCT of the left and right kidney were 0.74 cm (range 0.10-2.15 cm) and 0.75 cm (range 0.11-1.92 cm) respectively. The mean displacement of the left and right hemi-diaphragms were 1.34 cm (range 0.27-2.76 cm) and 1.45 cm (range 0.45-3.26 cm) respectively. The mean displacement of the anterior abdominal wall (abdomen) was 0.57 cm (range 0–1.06 cm) (Table 1).

**Table 1:** Descriptive statistics of measured organ displacement

**Parameter**	**Organ displacement (cm)**					
	***Left kidney***	***Right kidney***	***Left diaphragm***	***Right diaphragm***	***Abdomen***	***Liver dome (PDF)***
*Mean*	0.74	0.75	1.34	1.45	0.57	1.56
*Standard Dev*	0.44	0.40	0.50	0.55	0.25	0.53
*Median*	0.61	0.69	1.27	1.38	0.56	1.49
*Minimum*	0.10	0.11	0.27	0.45	0.00	0.74
*Maximum*	2.15	1.92	2.76	3.26	1.06	2.91
*Interquartile range*	0.45-0.98	0.49-0.97	1.07-1.56	1.08-1.77	0.41-0.74	1.17-1.85
*90th percentile*	1.33	1.30	2.12	2.21	0.98	2.33

**Figure 3:**
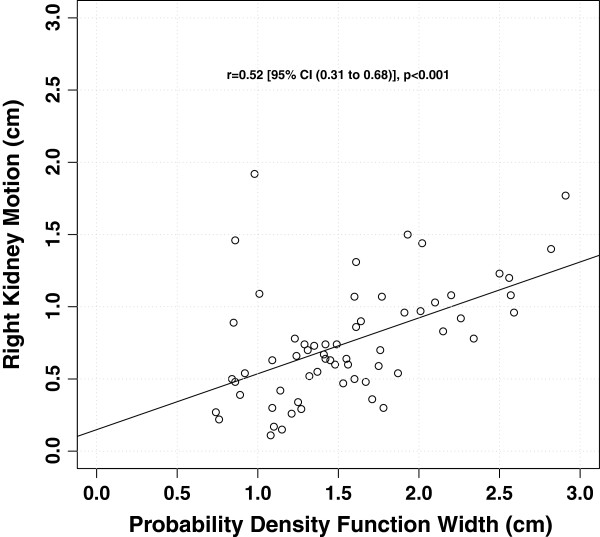
Relationship between pdf width and right kidney displacement.

**Figure 4:**
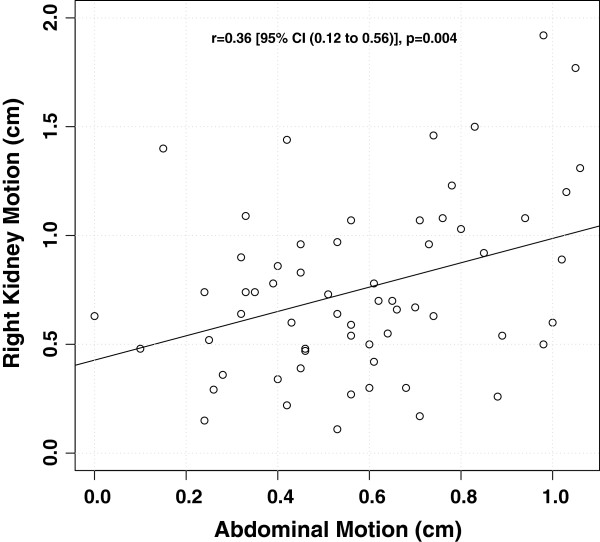
Relationship between abdominal displacement and right kidney displacement.

Both left and right hemi-diaphragm displacements were correlated with left and right kidney displacements respectively, with a slightly weaker correlation for the left kidney/left diaphragm (*r=*0.45, [95% CI 0.22 to 0.63], *p*=<0.001) than for the right kidney/right diaphragm (*r*=0.57, [95% CI 0.37 to 0.72], *p*=<0.001). The width of the PDF showed a statistically significant correlation with right kidney displacement (*r*=0.52 [95% CI 0.31 to 0.68], *p*<0.001) (Figure 3), but not with left kidney displacement (*r*=−0.08 [95% CI −0.33 to 0.17], *p=*0.796) nor abdominal wall displacement (*r*=−0.18 [95% CI −0.07 to 0.42], (*p=*0.151). There was a statistically significant correlation between the magnitude of right kidney displacement and that of abdominal displacement (*r*=0.36 [95% CI 0.12 to 0.57], *p*=0.004) (Figure 4). However the correlation between magnitude of left kidney displacement and abdominal wall displacement had only approached statistical significance (*r*=0.24 [95% CI −0.01 to 0.47], *p*=0.056). Age was not associated with either the left kidney (*r=*0.05 [95% CI −0.20 to 0.29], *p*=0.710) or right kidney (*r*=−0.08 [95% CI −0.33 to 0.17], *p*=0.517) displacement. The mean (±standard deviation) of left and right kidney motion was 0.95 cm (±0.63 cm) and 0.83 cm (±0.39 cm) respectively for patients with liver tumors and was 0.61 cm (±0.41 cm) and 0.74 cm (±0.40 cm) respectively for patients with thoracic tumors. The magnitude of left and right kidney displacement was not statistically different between patients with liver tumors and those with thoracic tumors (student’s t-test, *p=*0.257 and *p*=0.519 respectively). Similarly, tumor site (thoracic versus liver) did not affect abdominal displacement nor PDF measurement (*p*=0.259 and *p*=0.180 respectively).

**Figure 5:**
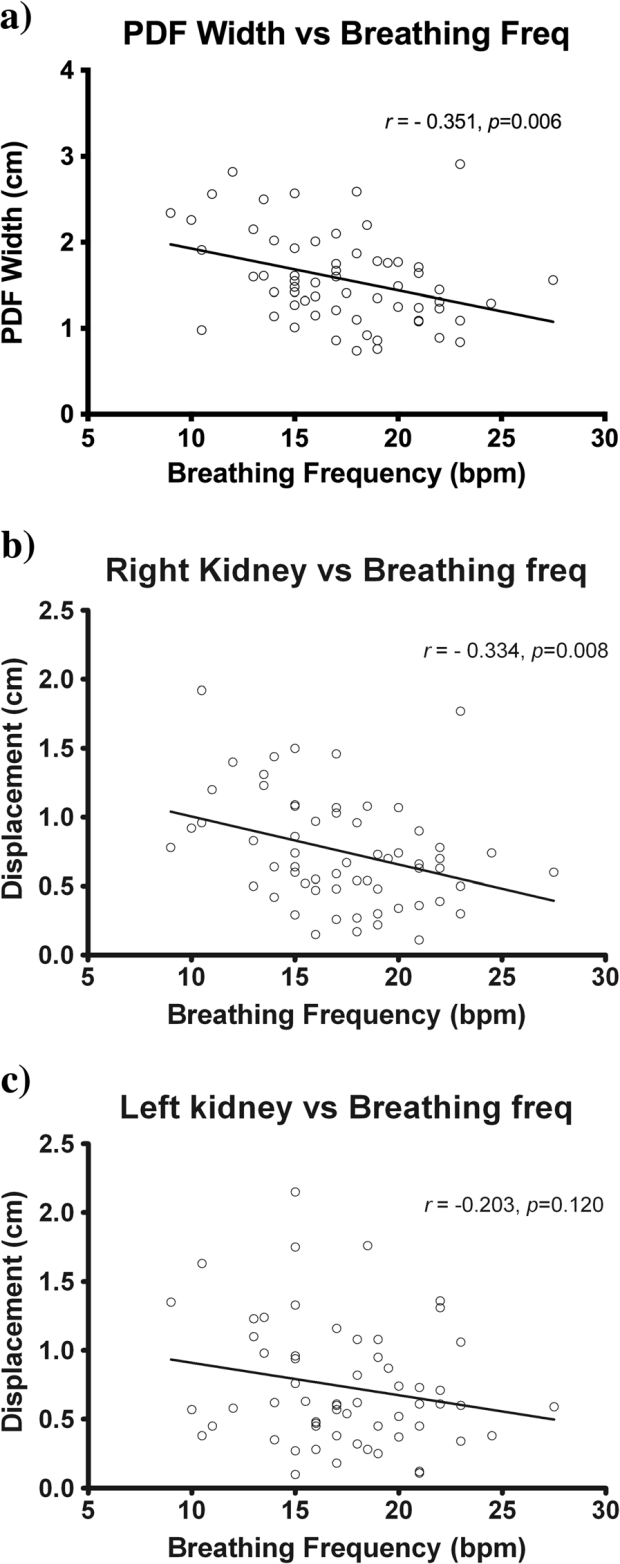
Relationship between patient average breathing frequency and magnitude of a) PDF, b) right kidney and c) left kidney.

The patient breathing frequency was recorded as a maximum, minimum and average in breaths per minute (bpm) from the breathing trace acquired at the time of the 4DCT. Of the 61 evaluable breathing traces, the mean (range) of the maximum, minimum and average breathing frequency was 20.1 (11–38) bpm, 15.1 (9–24) bpm and 17.3 (9–27.5) bpm respectively. The average breathing frequency showed a negative correlation with the magnitude of the PDF (*r=−*0.124, [95% CI −0.554 to −0.110, *p*=0.006) and the right kidney displacement (*r=−*0.112, [95% CI −0.541 to −0.090], *p*=0.008). However, breathing frequency was not correlated to left kidney displacement (*r*= −0.23, [95% CI −0.44 to 0.05], *p=*0.120) (Figure 5 a-c).

## Discussion

There is a resurgent interest in radiotherapy use for primary RCC since the advent of hypofractionated SABR techniques. Several treatment strategies have been developed to account for target motion, including respiratory gated delivery or delivery to the entire volume of tumor excursion. The latter strategy uses fields that incorporate the internal target volume (ITV), which encompasses the gross tumor volume in addition to an internal margin for tumor motion [4]. Understanding the amplitude of motion expected in either kidney is important in determining appropriate radiotherapy margins as typical SABR planning target volumes (PTVs) have a very narrow margin applied to the respective ITV, typically around 5 mm or less [7,8]. An understanding of a population average of motion is critical as breathing motion is complex, variable, and known to change over time at an individual level with transient instabilities [9]. Our population of patients showed a mean amplitude motion of 0.74 cm and 0.75 cm for both the left and right kidneys respectively, with 75 percent of patients having subcentimeter maximum kidney excursion. However, 10 percent of patients had greater than 1.33 cm and 1.30 cm of motion for the left and right kidneys respectively.

A consequence of the use of an ITV concept for treatment (as opposed to gated radiotherapy delivery) is an increase in the amount of normal tissue irradiated in order to account for tumor motion. In the context of the kidney however, this motion relates to a relatively large organ typically measuring 3 cm by 6 cm by 12 cm [10]. The kidney is an organ that functions in a radiobiological sense in a parallel rather than serial fashion for the expression of radiation toxicity such as hypertension or loss of renal function. A relatively small respiratory induced organ motion in the context of a large parallel functioning volume may mitigate the consequences of non-gated treatment delivery. This view is reinforced by historically low risks of clinical toxicity after SABR for renal cell carcinoma. Interestingly, symptomatic kidney injury has not been reported after SABR to kidney targets to date. The QUANTEC consensus group publication [11] suggests that “one hypothesis is that nearly complete sparing of a substantial volume of the kidney should be associated with compensatory effects and preservation of renal function, despite the delivery of focal high doses”. The low risk of clinical toxicity after SABR to the kidney has been most eloquently researched in a study published by Svedman et al. [12]. This study assessed the effect of SABR in patients with only one functioning kidney with up to 6-years of follow up. Five of the seven patients had no change in renal function. Two patients had mild asymptomatic increases in renal function, without the need for medical or dialysis intervention. None of the patients developed hypertension. In the context of low clinical risk of renal toxicity, and given that the large majority of patients in our study had subcentimeter kidney motion, our group suggests that SABR delivery with an ITV concept may be a reasonable strategy for the majority of these patients. However, in select patients with large respiratory excursion, strategies to reduce the internal tumor motion (such as respiratory training [13] or abdominal compression [14]) may be warranted to reduce the integral dose to surrounding normal tissue.

This study presents the largest series to date assessing patient kidney motion. Previous smaller studies have used a variety of different imaging modalities under varying breathing conditions. These studies have demonstrated a broad range of values for kidney displacement. An early study demonstrated in 14 patients that kidney motion measured by radiographs under forced deep breathing conditions ranged between 0.1-3.2 cm and 0.3-2.1 cm in the right and left kidneys respectively [15]. Moerland et al. [16] found that forced breathing can induce movement as large as 6.6 cm in the left kidney and 8.6 cm in the right kidney in a study using magnetic resonance imaging (MRI). Under free breathing conditions using MRI in 12 patients, Bussels et al. [17] demonstrated a mean cranio-caudal displacement of the right kidney and left kidney of 1.61 cm and 1.69 cm respectively. A study by Wysocka et al. [18] using serial non-gated CTs in 22 patients treated for gastric cancer showed a median (range) free breathing cranio-caudal displacement of 0.6 cm (0–3.7 cm) and 0.8 cm (0.2–3.5 cm) in the left and right kidneys respectively, and a displacement of 0.6 cm (0–2.8 cm) for the diaphragm. Kim et al. [19] studied 9 healthy volunteers with 4DCT scans in the supine position showed mean kidney motion of 1.2 cm and mean hepatic dome motion of 1.7 cm. The largest report of kidney motion using 4DCT prior to our study was from Van Sörnsen de Koste [20] in an investigation of 54 patients, 49 with lung tumors and 5 with liver tumors. Mean (range) cranio-caudal mobility was observed to be 0.98 cm (0.25-3.00 cm) for the left kidney and 0.9 cm (0.25-2.05 cm) for the right kidney. Similarly, our series demonstrated a similar mean (range) of displacement for both the left and right kidney, at 0.74 cm (0.10-2.15 cm) and 0.75 cm (0.11-1.92 cm) respectively.

The use of the PDF for assessment of liver dome motion from the *average* reconstruction of the planning 4DCT is a relatively novel technique. We have extrapolated this technique from that described by Guckenberger et al. [6] to interpret respiratory excursion of the liver on CBCT) Our own group’s experience within the context of the FASTRACK prospective clinical trial (*ClinicalTrials.gov Identifier*: NCT01676428) indicates that kidney tumor image guidance can be particularly challenging when using online 3D CBCT techniques, particularly without the aid of implanted fiducials or contrast agents. During the treatment delivery workflow, should the kidney and tumor ITV not match to pre-treatment estimates, confirmation of the appropriateness of the ITV margin selected can be theoretically achieved through measurement of surrogate organ displacement. This present study suggests that the PDF width has only moderate correlation with right kidney displacement (*r*=0.52, *p*<0.001) and no correlation with the left kidney (*p=*0.151). Based on these results, the PDF width does not allow for reliable matching of respiratory induced kidney displacement. On a practical level, our study suggests that liver dome excursion should not be used as a surrogate for kidney target displacement as has been previously reported for liver targets [6].

In our study population there was a correlation between abdominal wall motion and right kidney displacement (*p*=0.004), however no significant correlation between abdominal motion and left kidney displacement (*p*=0.056). A similar de-coupling effect of breathing and organ motion was demonstrated for the left kidney in our study when assessing for patient breathing frequency. The breathing frequency was inversely correlated to the PDF and right kidney motion (*p*=0.006 and *p*=0.008 respectively), confirming the intuitive assumption that patients with more shallow, rapid breathing patterns have smaller respiratory induced organ excursion. In contrast, breathing frequency did not correlate with left kidney motion (*p*=0.120). Our hypothesis for this phenomenon is that the intimate association of the liver may constrain kidney motion on the right, whereas in contrast the left kidney largely floats within the perinephric fat, bounded only by Gerota’s fascia. Therefore the left kidney motion and relationship with surrounding upper abdominal organs may not be as rigidly conformal as the right kidney. On a practical level, this dissociation raises the concern that current evidence supporting the use of external surrogates for indirect tumor matching in lung and liver tumors may not be directly applicable to the context of SABR kidney treatments. This is an area in need of further investigation. The weak inter-patient correlation noted in this study, despite the large sample size, indicates that abdominal marker displacement is also an inadequate surrogate for kidney displacement.

A potential weakness of this study is that the 4DCT datasets are acquired for each scan over a relatively short time in the context of the clinical treatment times required for SABR kidney. Future studies utilizing long acquisition scanning techniques with high spatial resolution (such as cine MRI) or repeated 4DCTs would be necessary to fully elucidate the patterns of motion of upper abdominal organs over periods that more closely match that of treatment delivery. Baseline shifts of the liver and kidney displacement have been observed with a median shift of 6.5 mm and 6.6 mm respectively over longer breathing periods (Wysocka 2010). A baseline shift in patient breathing can potentially confound the verification of the PDF width. A 4D image guidance protocol to verify breathing motion should take this into consideration. For example, a 3D CBCT of the diaphragm dome can be matched to bony anatomy of the planning CT before assessment of the PDF width. Verification of the location of the maximum inspiration and expiration of the liver dome as well as its magnitude can ensure that a large baseline shift has not occurred. The use of planar imaging can also allow the entire range of diaphragm motion to be observed in order to verify displacement as well as maximum inspiration and expiration positions. However in a free breathing protocol, our clinic has experienced technical limitations in using fluoroscopic imaging in this context due to user variability in the measurement of liver dome displacement. For the present time the PDF width still serves as a useful tool to compare the patient’s depth of respiration on the planning CT and the pre-treatment CBCT.

## Conclusion

Kidney motion during respiration for the majority of patients is subcentimeter in magnitude and justifies the use of an ITV for treatment delivery. In a small minority kidney motion can be significant and poses a potential technical limitation in the delivery of stereotactic image guided radiotherapy. Neither the abdominal wall nor the liver dome were found to be adequate surrogates of kidney displacement in our study, and diaphragmatic displacement was only moderately correlated. Therefore, these organs should not be used to estimate the appropriateness of kidney ITV margins unless validated in an individual patient.

## Consent

Written informed consent was obtained from the patient for the publication of this report and any accompanying images.

## Competing interests

The authors declare that they have no competing interest.

## Authors’ contributions

SS is the primary author of this research, and was responsible for the project. DP provided intellectual input to study design and collected data for the study. MB contributed to study design and provided statistical analysis for the study. SG, KD, TD, FF and TK contributed to study design and manuscript preparation. FF and TK were responsible for team co-ordination and study oversight. All authors read and approved the final manuscript.
